# All-trans retinoic acid attenuates airway inflammation by inhibiting Th2 and Th17 response in experimental allergic asthma

**DOI:** 10.1186/1471-2172-14-28

**Published:** 2013-06-22

**Authors:** Jinhong Wu, Yanjie Zhang, Qi Liu, Wenwei Zhong, Zhenwei Xia

**Affiliations:** 1 Department of Pediatrics, Ruijin Hospital Affiliated to Shanghai Jiao Tong University School of Medicine, Ruijin 2nd Road 197, Shanghai 200025, China

**Keywords:** Asthma, All-trans retinoic acid, Th2, Th17, Regulatory T cells

## Abstract

**Background:**

Airway inflammation is mainly mediated by T helper 2 cells (Th2) that characteristically produce interleukin (IL)-4, IL-5, and IL-13. Epidemiological studies have revealed an inverse association between the dietary intake of vitamin A and the occurrence of asthma. Serum vitamin A concentrations are significantly lower in asthmatic subjects than in healthy control subjects. It has been reported that all-trans retinoic acid (ATRA), a potent derivative of vitamin A, regulates immune responses. However, its role in Th2-mediated airway inflammation remains unclear. We investigated the effects of ATRA in a mouse model of allergic airway inflammation.

**Results:**

We found that ATRA treatment attenuated airway inflammation and decreased mRNA levels of Th2- and Th17-related transcription factors. The data showed that airway inflammation coincided with levels of Th2- and Th17-related cytokines. We also showed that ATRA inhibited Th17 and promoted inducible regulatory T-cell differentiation, whereas it did not induce an obvious effect on Th2 differentiation in vitro. Our data suggest that ATRA may interfere with the in vivo Th2 responses via T-cell extrinsic mechanisms.

**Conclusions:**

Administration of ATRA dramatically attenuated airway inflammation by inhibiting Th2 and Th17 differentiation and/or functions. ATRA may have potential therapeutic effects for airway inflammation in asthmatic patients.

## Background

Epidemiological studies have reported an inverse association between asthma and the intake of vitamin A. Dietary vitamin-A intake and serum vitamin-A concentrations are significantly lower in patients with asthma than in healthy control subjects or in people with severe asthma than in those with mild asthma [1-3]. All-trans retinoic acid (ATRA) is a biologically active metabolite of vitamin A with profound effects on T-cell activation [4,5], differentiation [6], and function [7,8]. ATRA binds to retinoic acid receptors in the nucleus leading to the activation of transcription of many target genes [9-11]. Emerging evidence demonstrates that ATRA signaling is critical for T cells differentiation and function. ATRA is an early mediator in the development of CD4^+^ T cell-mediated immunity, and also plays a pivotal role in optimal effector and effector memory CD8^+^ T cell differentiation in which vitamin A supplementation is used to augment effector responses [12,13]. Meanwhile, ATRA promotes Foxp3^+^ regulatory T-cell (Treg) differentiation and sustains the stability and function of natural Tregs in an inflammatory milieu. In addition, it suppresses Th17 differentiation. ATRA and rapamycin can synergistically increase TGF-β-mediated Foxp3 induction in antigen-specific Th2 memory cells when effector cytokines are neutralized. These Foxp3^+^ T cells converted from Th2 memory cells possess Treg activity and can ameliorate Th2 memory-mediated airway hyperreactivity and eosinophilic inflammation [14-20]. Moreover, ATRA suppresses Th2-related chemokine expression in vitro by down-regulating the expression of interleukin (IL)-5 receptor and inhibiting eosinophil and basophil differentiation [21-23]. In addition to modulating T cells, ATRA plays an important role in the maintenance of the normal epithelial mucociliary phenotype [21]. However, the role of ATRA on airway inflammation after allergenic challenge has not been established. The goal of this study is to determine whether ATRA alters Th2 response to modulate the severity of airway inflammation in an ovalbumin (OVA)-induced allergic airway inflammation animal model.

## Results

### Administration of ATRA attenuated lung inflammation

**Figure 1:**
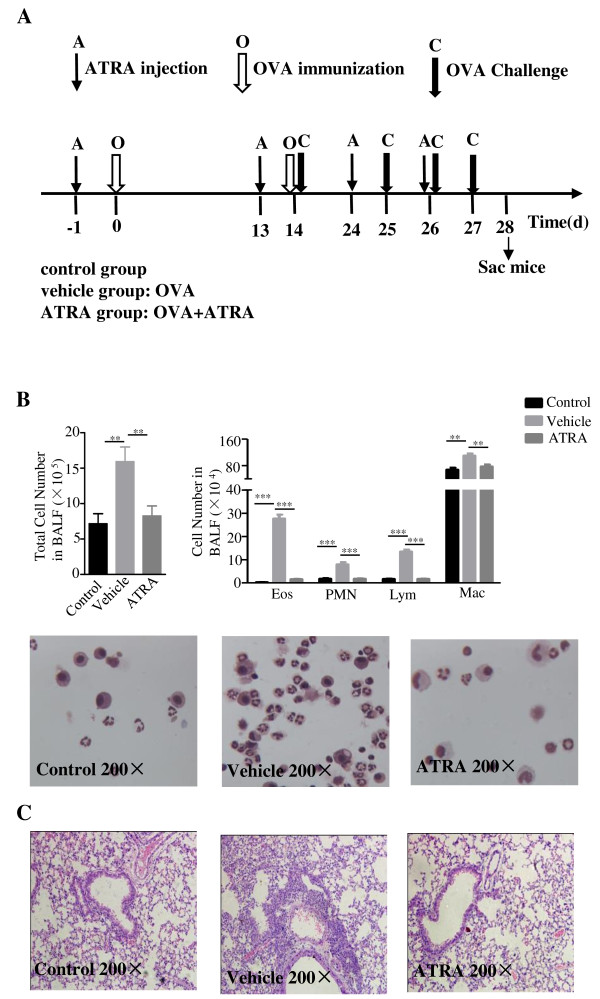
**ATRA-attenuated lung inflammation.** Mice received i.p. injection of 400 μg/mouse ATRA or vehicle on days -1, 13, 24, and 26, which is 1 day prior to OVA sensitization, while mice were injected with PBS as control. **A**. The protocol used to evaluate the effects of ATRA on asthma manifestations in vivo. **B**. BALF from the mice was analyzed 24 h after the final challenge (day 28). Results are shown as the total number of cells in BAL fluid. Total, total cell number; Mac, monocyte/macrophage; Eos, eosinophils; Lym, lymphocytes; PMN, neutrophils. Values expressed as number of cells × 10^5^/ml or 10^4^/ml are means ± SEM (n = 10 in each group; **, p < 0.01). Data are representative of 3 independent experiments. **C**. Histopathological analyses on lung tissues from three groups at 24 hours after the final challenge (day 28). Mice were sacrificed at 24 hours after the final challenge, lung tissues were stained with H&E, and the inflammatory cells were analyzed by light microscopy (magnification: 200×). An increased number of eosinophils was observed in the vehicle group compared with the control group, while such an increase was obviously attenuated in the ATRA group. Data are representative of 10 mice in each group of 3 independent experiments.

Clinical studies have shown that dietary vitamin A intake and serum vitamin A concentrations are significantly lower in patients with asthma than in healthy control subjects, implicating that vitamin A might be an attractive candidate for asthma treatment. ATRA is a biologically active metabolite of vitamin A [1-3]. To determine the effects of ATRA on allergic airway inflammation, an OVA-sensitized murine airway inflammation model was used. The ATRA group received an intraperitoneal (i.p.) injection of 400 μg/mouse of ATRA. The vehicle group was injected by corn oil (vehicle group). All treatments were administrated prior to OVA sensitization. A control group of mice without OVA sensitization was injected with PBS. Mice were sacrificed and lung inflammation was evaluated at 24 hours after the final challenge (day 28). As shown in Figure 1, OVA treatment in the vehicle group induced a substantially increase of total cells, eosinophils, neutrophils, lymphocytes and macrophages counts in bronchoalveolar lavage fluid (BALF) as compared with the control group (Figure 1A), while a significant decrease was observed after ATRA intervention (Figure 1B, **, *p* < 0.05; ***, *p* < 0.01). Observation by light-microscopy confirmed that OVA priming and activation led to a marked peribronchial leukocyte, especially eosinophilic infiltration. More importantly, the inflammatory response was significantly attenuated after treatment with ATRA (Figure 1C). These data indicated that ATRA treatment mitigated the inflammatory responses in the antigen-induced allergic process.

### Administration of ATRA decreased the levels of Th2 and Th17-related cytokines, and down-regulated the expression of corresponding transcription factors in the lung

**Figure 2:**
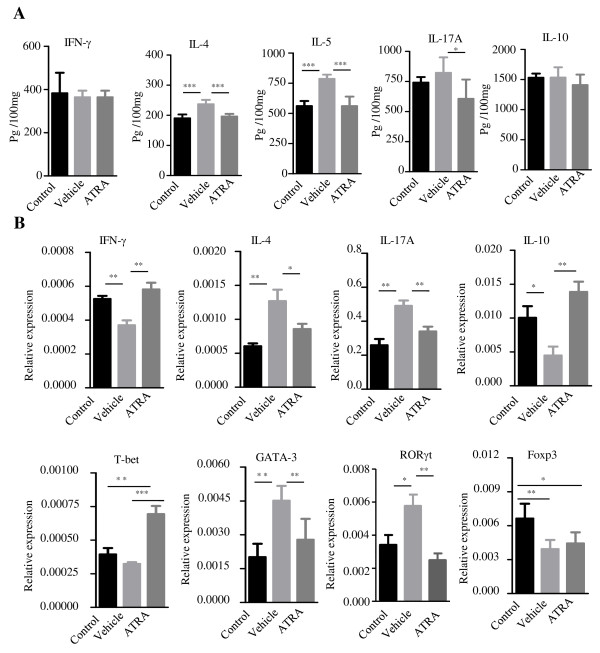
**Administration of ATRA decreased the levels of Th2- and Th17-related cytokines and down-regulated the expression of transcription factors in the lung. A**. The levels of IL-4, IL-5, IFN-γ, IL-10, and IL-17 in the lung homogenates were assessed by ELISA. Compared with the control group, the levels of IL-4 and IL-5 in the lung significantly elevated in vehicle group, but ATRA significantly reduced IL-4, IL-5 levels and slightly decreased the IL-7A level, but there was no significant difference for the levels of IFN-γ or IL-10 in different groups. **B**. Treatment with OVA up-regulated the expression of IL-4, IL-17A mRNA in lung; ATRA suppressed these cytokine transcription levels and increased IFN-γ and T-bet mRNA expression. Meanwhile, ATRA moderately increased the IL-10 mRNA level and had no effect on Foxp3 mRNA transcription in vivo. Data represent the mean ± SEM with n = 10 for each group (***, *p* < 0.001; **, *p* < 0.01; *, *p* < 0.05). Data represent two independent experiments.

To determine the effects of ATRA on Th cells and the cytokines produced by these cells after allergen stimulation in the lung, IL-4, IL-5, IFN-γ, IL-10, and IL-17 levels in the lung homogenates were assessed by ELISA. The levels of IL-4 and IL-5 in the vehicle group were significantly elevated compared with those in the control group (***, *p* < 0.001). ATRA pretreatment significantly reduced the IL-4 and IL-5 levels and slightly decreased the IL-17A level, but there was no significant difference on the levels of IFN-γ or IL-10 in the compared groups (Figure 2A). Meanwhile, real-time PCR results showed that mRNA levels of IL-4 and IL-17A in the lung were consistent with the ELISA results. Furthermore, the mRNA levels of GATA-3 and RORγt were significantly decreased in the ATRA group compared with the vehicle group (*p* < 0.01), while the expression of the IFN-γ and Th1-related transcription factor T-bet was much higher in the ATRA group than in the vehicle group. In contrast with the previous in vitro results, ATRA moderately increased the IL-10 mRNA level and had no effect on the expression of the Treg-related transcription factor Foxp3 in the lung (*, *p* < 0.05, Figure 2B).

### Administration of ATRA suppressed the levels of Th2 and Th17-related cytokines in draining lymph nodes

**Figure 3:**
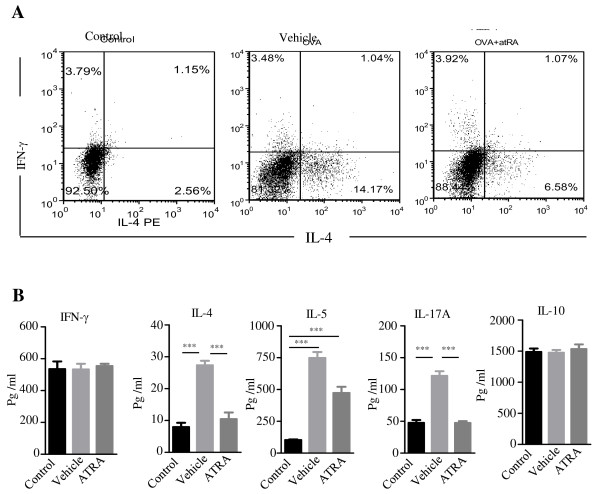
**Administration of ATRA suppressed the levels of Th2- and Th17-related cytokines in draining lymph nodes. A**. Mice were sacrificed at 24 hours after the final challenge and lymphocytes were isolated from mediastinal lymph nodes and stimulated with OVA_323–339_ peptide for 72 hours. The percentages of IL-4 positive and IFN-γ positive CD4 T cells were analyzed by FACS. The results showed that ATRA pretreatment decreased the percentages of IL-4 positive T cells compared with the vehicle mice. **B**. The concentration of IL-4, IL-5, IL-10, and IL-17A in supernatants of cultured mediastinal lymph nodes were detected by ELISA. The levels of IL-4, IL-5, and IL-17A were significantly decreased after pretreatment with ATRA. There was no significant difference in IFN-γ and IL-10 among the three groups. Data represent the mean ± SEM with n = 10 for each group (***, *p* < 0.001). Data are representative of 3 independent experiments.

To further determine the suppressive effect of ATRA in airway inflammation, we examined Th1, Th2, and Th17 responses in draining lymph node cells on day 28. Lymphocytes were isolated from mediastinal lymph nodes and stimulated with OVA_323–339_ peptide for 72 hours. The percentages of IL-4 and IFN-γ positive CD4 T cells were analyzed by FACS. The results showed that the number of IL-4-positive T cells significantly increased in the vehicle group compared with the control group. ATRA pretreatment decreased the percentages of IL-4-positive T cells compared with the mice treated with vehicle alone. However, there was no significant difference in the percentages of IFN-γ-positive CD4 T cells between the vehicle and the ATRA group (Figure 3A). To analyze the effects of ATRA on CD4 T-cell function, supernatants from lymphocytes stimulated with OVA_323-339_ peptide were analyzed with ELISA. Compared with the control mice, the levels of IL-4, IL-5, and IL-17A were significantly increased in the vehicle mice, however, these cytokines were dramatically decreased after pretreatment with ATRA (**, *p* < 0.001). There was no significant difference in IFN-γ and IL-10 among the three groups (Figure 3B).

### In-vivo ATRA treatment inhibited Ag-specific Th2 responses with no obvious effect on Foxp3^+^ Treg population in the spleen

**Figure 4:**
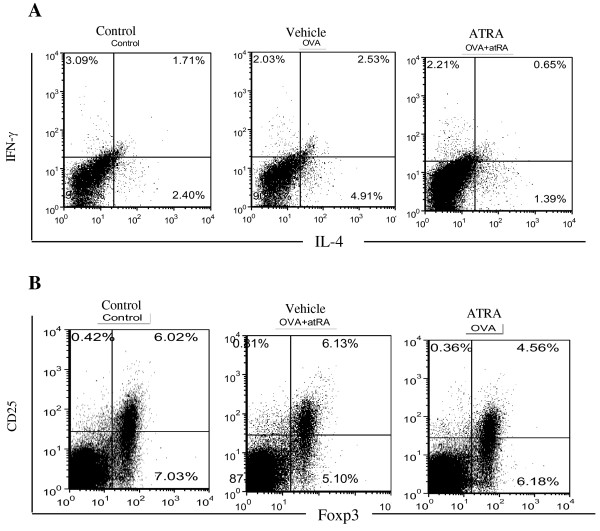
**ATRA treatment inhibited Ag-specific Th2-cell responses and had no effect on Foxp3**^**+ **^**Treg population in spleen.** Mice were sacrificed at 24 hours after the final challenge. **A**. Spleenocytes were isolated from spleens and stimulated with OVA_323–339_ peptide for 72 hours, then intracellular stained with indicated antibodies after stained with the extracellular maker CD4. The percentages of IL-4 and IFN-γ positive CD4 T cells in the spleen were analyzed by FACS. **B**. Spleenocytes were isolated from spleens stained for the extracellular maker CD4, CD25 and intracellular markers Foxp3 and analyzed by FACS. The percentages of Treg cells (CD25^+^ Foxp3^+^) were gated on CD4^+^ T cells, The results showed that ATRA pretreatment decreased the percentages of IL-4 positive T cells but no effect on Treg cell population. Data represent five mice of each group in three independent experiments.

Furthermore, in addition to draining lymph nodes, splenic Th-cell populations were examined for the effects of ATRA treatment. Splenocytes were stimulated with OVA_323–339_ peptide for 72 hours and then intracellularly stained with anti-IL-4 and IFN-γ antibodies. The percentages of IL-4 and IFN-γ in gated CD4 T cells in the spleen were analyzed by FACS. The results showed that the percentages of IL-4-positive T cells were significantly increased in the vehicle group compared with the control group, while the percentages of IFN-γ positive CD4 T cells were slightly decreased in the vehicle and the ATRA treated groups compared with the control group (Figure 4A). To examine whether ATRA treatment could increase the Foxp3^+^ Treg population in vivo, splenocytes were stained for Foxp3 and CD25 and analyzed by FACS. Unlike the effect of ATRA on conventional Foxp3^-^ CD4 T cells, ATRA treatment did not increase the Foxp3^+^ Treg population in the spleen of immunized mice. These results showed that ATRA pretreatment decreased the percentage of IL-4-positive T cells without obvious effects on the Treg population in the spleen (Figure 4B).

### Retinoic acid does not obviously affect Th2 differentiation in vitro

**Figure 5:**
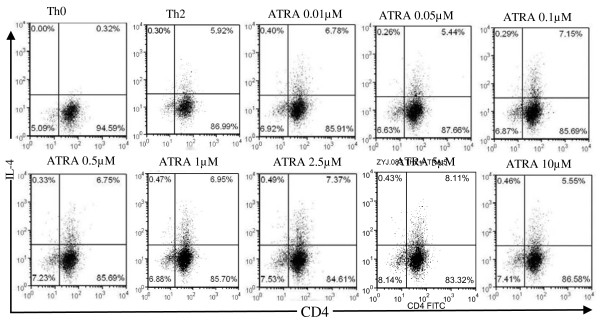
**Retinoic acid does not affect Th2 cells differentiation in vitro.** Naïve CD4^+^ T cells (CD4^+^ CD62L^hi^ CD25^-^) were purified from DO11.10 mice by FACS. Isolated CD4^+^ CD62L^+^T cells were stimulated with OVA peptide _323–339_ in Th2 skewing condition with/without different concentration ATRA (0.01-10 μmol/L) for 3 days. The cells were then stained with CD4 and IL-4 antibodies and analyzed by FACS. Dot-plot pattern showed intracellular cytokine staining. Data are representative of 3 independent experiments.

To explore whether in-vivo-reduced Th2 cytokines following ATRA treatment was directly influenced by ATRA, we assessed the effect of ATRA on Th2 differentiation in vitro. Naïve CD4^+^CD62L^+^ T cells from DO11.10 mice were cultured under a Th2-skewing condition without or with different concentrations of ATRA. After the cells were cultured for 5 days, IL-4 expression was determined by intracellular staining. Similar percentages of IL-4-producing cells were detected in CD4 T cells with or without ATRA treatment (Figure 5), suggesting that ATRA might not influence Th2 differentiation in vitro.

## Discussion

Previous reports showed that ATRA is the biological active metabolite of vitamin A and has an important immunomodulatory effect by inhibiting Th17 polarization and enhancing Foxp3 expression [14-16]. By using a murine Th2-mediated airway inflammation model, we demonstrated that the administration of ATRA attenuated OVA-induced airway inflammation by decreasing Th2 and Th17 related cytokines and inflammatory cells in the airway and ATRA mediated reduction of Th2 cytokines was via inhibiting GATA-3 expression. Our findings provide further supports for the anti-inflammatory role of ATRA in the treatment of lung diseases.

OVA-induced allergic asthma is recognized as a pathological condition that results from chronic airway inflammation characteristically associated with the infiltration of lymphocytes, eosinophils, macrophages, and neutrophils into the bronchial lumen. An aberrant Th2-type response to allergens is characterized by the overproduction of IL-4, IL-5, and IL-13, which are critical for the maintenance of ongoing IgE-mediated eosinophilic inflammation [24-26]. Accumulating evidence has suggested that aberrant IL-17 production is a key determinant of severe forms of asthma. IL-17A induces lung-structural cells to secrete proinflammatory cytokines (TNF, IL-1β, granulocyte colony-stimulating factor, and IL-6) and chemokines (CXCL1, CXCL2, and CXCL8/IL-8), thereby triggering neutrophil infiltration [27-29].

We showed that the administration of ATRA inhibited OVA mediated secretion of IL-4 and IL-5 in the lung, draining lymph nodes, and the spleen. Consistent with the decreased levels of Th2-related cytokines, the recruitment of inflammatory cells, especially eosinophils, neutrophils, lymphocytes, and macrophages, was markedly decreased in the BALF and the lung after ATRA administration. These results indicated that ATRA may alleviate airway inflammation by reducing Th2 cytokines. Meanwhile, T-bet and GATA-3 are responsible for the regulation of the cytokine genes during Th1/Th2 differentiation. GATA-3 has been shown to promote the expression of several Th2 cytokines, including IL-4, IL-5, and IL-13 [30,31]. It is well known that overexpression of GATA-3 predisposes for Th2-mediated diseases such as allergic asthma and suppression of GATA-3 expression in the lung reduces IL-4, IL-5, and IL-13 productions concurrently [30,31]. Compared with the vehicle group, the GATA-3 mRNA level in the lung in the ATRA treated group was significantly decreased, which may partially explain the reduction of IL-4 and IL-5 production in these mice. In addition, a recent study performed in chronic asthma model showed that prolonged ATRA treatment tends to inhibit Th17 cell infiltration and neutrophilia without obvious alteration of Th2 cell numbers [32]. The different observations are probably due to different Th-dominate asthma models used between the two studies, in which the immune responses are mediated by different Th subsets.

The concentration of retinoic acid is likely to be critical to its effect on the airway responses. Previous studies have demonstrated that ATRA reverses the airway hyperresponsiveness (AHR) and exogenous administration of retinoic acid is capable of attenuating the asthma phenotype [32-34]. However, it has also been reported that ATRA promotes Th2 development to exacerbate allergic immune and inflammatory responses during systemic sensitization [35]. The differences in the responses of the airway are probably linked to the different concentrations and time point used in those studies. Indeed, the study performed by Mateu et al. supports the above notion. They have found that retinoic acid directly enhances allergic responses in vivo, but higher doses effectively decrease AHR by inhibiting IL-5 production [36]. Therefore, the concentration and time point of retinoic acid should be carefully considered in the applications.

Of note, ATRA was not able to affect Th2 differentiation in a Th2 skewing condition in vitro. The discrepancy between the effects of ATRA on in-vitro and in-vivo Th2 responses suggested that ATRA might not intrinsically control Th2 differentiation. Rather, it might do so by inhibiting the Th17 response or by modulating the function of antigen presenting cells. In addition, Bidad K et al. have reported that ATRA can significantly decrease Th17 cells in patients with ankylosing spondylitis. The effect of ATRA in such patients serves as an immunomodulator on deviated immune cells, which is associated with decreased inflammatory cytokine TNF-α secretion [36]. In our study, we also found that the level of IL-17A was slightly decreased in the draining lymph nodes and lung, while IFN-γ and T-bet mRNAs were slightly increased in the lung after the administration of ATRA. Unlike the in-vitro results previously reported by Elias et al. [15], our in vivo data showed that ATRA moderately increased IL-10 expression without affecting IL-10 production, Foxp3 expression and Treg numbers in the lung or spleen. The different responses of Treg cells might be attributed to the difference between the in-vivo environment and in-vitro condition [32]. Additionally, ATRA effects on Treg cells in vivo might be also influenced by the exposure time of ATRA. For example, Zhao et al. found that the proportion of Foxp3^+^ CD4 Treg cells in the lymph nodes is temporarily increased after a week of ATRA treatment [32]. The data suggest that ATRA may affect Treg cells in a context- and time-dependent manner.

## Conclusions

In summary, ATRA administration significantly decreased Th2- and Th17-related cytokines and markedly reduced airway inflammation in a murine allergic airway inflammation model. These findings suggested that ATRA may serve as an effective therapy for allergic airway inflammation. Our study suggested potential benefits of Vitamin-A supplement for asthma patients and may provide the basis for further investigation of the mechanisms underlying the potential therapeutic effects of ATRA or vitamin A in controlling the airway inflammation of asthma.

## Methods

### Animals

A total of 90 female BALB/c mice and 3 DO11.10 mice at 6 ~ 8 weeks of age were purchased from the Shanghai SLAC Laboratory Animal Company. All mice were maintained under specific pathogen-free conditions in our animal facility. BALB/c mice were randomly divided into 3 groups: a control group, OVA plus vehicle (vehicle group), and OVA-plus ATRA (ATRA group). Each group included 10 mice, and 3 independent experiments were performed. Animal experiments were performed according to the Ethics Committee of Ruijin Hospital, Shanghai Jiaotong University School of Medicine.

### Ag sensitization and challenge protocol and administration of ATRA

The mouse-asthmatic model was established as described previously [37,38]. In both vehicle and ATRA groups, mice received i.p. injections of 100 μg OVA (Sigma-Aldrich) in 0.2 ml Al(OH)_3_ adjuvant suspension on days 0 and 14. On days 14, 25, 26, and 27, mice were anesthethized with isoflurane and intranasally received 100 μg of OVA in 0.05 ml phosphate buffer saline (PBS) (days 14) and 50 μg of OVA in 0.05 ml PBS (days 25, 26, and 27). The control group was sensitized with the same volume of Al(OH)_3_ and challenged with normal saline instead of OVA. ATRA was dissolved in dimethyl sulfoxide (DMSO) and diluted in corn oil. Mice in the ATRA group received i.p. injections of 400 μg ATRA on days –1, 13, 24, and 26, the latter being one day prior to OVA sensitization. The mice in the vehicle group were injected with the same volume of corn oil.

### Bronchoalveolar lavage fluid (BALF)

Twenty-four hours after the final challenge (day 28), BALB/c mice were sacrificed by CO_2_. BALF was obtained by the slow injection of 0.4 ml ice-cold PBS into the trachea using a 22-inch i.v. with cathetering three times (total 1.2 ml). This procedure recovered 80 to 90% of the infused fluid. The total number of cells in BALF was counted using a hemacytometer. After cytospin, the cells were fixed and stained by hematoxylin-eosin (H&E). A total of 200 cells were randomly selected to calculate eosinophils, neutrophils, lymphocytes and macrophages under the microscope (Olympus AX70, Japan). Different cell counts were calculated by the following equation:

$$\begin{array}{rcl} \mathrm{Total}\;\mathrm{number} & = & \left(\mathrm{number}\;\mathrm{of}\;\mathrm{target}\;\mathrm{cell}\;\mathrm{under}\;\mathrm{the}\right)\\ & & \left(\mathrm{microscope}/200\right)\;\times \;\mathrm{total}\;\mathrm{cell}\;\mathrm{count} \end{array}$$

### Histology

The lung lobe was fixed in 10% formalin, embedded in paraffin, and sectioned in 4 μm slices. Sections were stained with H&E and examined microscopically.

### Detection of soluble cytokine levels in lung

To determine soluble cytokine levels in the lungs, a snap-frozen right lung lobe from each mouse was thawed on ice and homogenized in a buffer containing a protein-protease inhibitor and 0.1% Triton X-100. The samples were centrifuged, and 50 μl of cell-free supernatant was analyzed by ELISA according to the manufacturer’s instructions. Cytokine levels including IL-4, IL-5, interferon (IFN)-γ, IL-10 and IL-17 in the culture supernatants of mediastinal lymph nodes, splenocytes and lung homogenates were quantified. All the antibodies for the assay were purchased from Biolegend.

### Cells stimulation and cytokines assay in vitro

Lymphocytes and splenocytes were used to determine the immune-regulatory effects of ATRA. On day 28, the mice were sacrificed and mediastinal lymph nodes and spleens were isolated. The cell clumps were disaggregated into single-cell suspensions using nylon mesh (30 μm) filtration. Red blood cells were removed by a red-blood-cell lysis buffer. The isolated lymphocytes and splenocytes were cultured in a 0.5 ml RPMI 1640 medium (Sigma-Aldrich) supplemented with 2 mmol/L L-glutamine, 10% fetal calf serum (FCS), 100 mg/ml streptomycin, 100 IU/ml penicillin, 10 mm HEPES, and 20 mm sodium hydrogen carbonate (Invitrogen Life Technologies, Carlsbad, California, USA). Lymphocytes and splenocytes were cultured at the density of 2 × 10^6^/ml in 48 well plates under the stimulation with 10 μg/ml OVA_323–339_ peptide for 72 hours. The cells were harvested and intracellular staining for IFN-γ, IL-4, and IL-17A were performed for flow cytometry. The cell supernatants were collected and analyzed for IFN-γ, IL-4, IL-5, IL-17A, and IL-10 using mouse ELISA kits (Biolegend).

### Real-time PCR analysis

For the total RNA isolation, lung tissue was removed from the animal, immediately froze in liquid N_2_, and stored at –70°C for assaying. The frozen lung tissue was then homogenized in TRIzol reagent (Invitrogen Life Technologies, Carlsbad, California, USA), and the total RNA was isolated according to the manufacturer’s instructions. Reverse transcription was performed using 40 ng of the total RNA with Superscript III RT kit (Qiagen) and oligo(dT) primers (Invitrogen Life Technologies, Carlsbad, California, USA) as recommended by the manufacturer. The Bio-Rad quantitative PCR SYBR Green Master Mix (Bio-Rad Laboratories) was used and performed the following program: 95°C for 5 min and 40 cycles of amplification at 95°C for 15 seconds and 58°C for 60 seconds. Relative levels of target mRNA were compared with β-actin using the 2^−ΔΔCt^ method. Primer sequences were obtained from Integrated DNA Technologies. Sequences were as follows: β-actin Forward: 5′-GGC TGT ATT CCC CTC CAT CG-3′, Reverse: 5′-CCA GTT GGT AAC AAT GCC ATG T-3′; IL-4 Forward: 5′-GGT CTC AAC CCC CAG CTA GT-3′, Reverse: 5′-GCC GAT GAT CTC TCT CAA GTG AT-3′; IL-10 Forward: 5′-GCT CTT ACT GAC TGG CAT GAG-3′, Reverse: 5′-CGC AGC TCT AGG AGC ATG TG-3′; IL-17A Forward: 5′-TTT AAC TCC CTT GGC GCA AAA-3′, Reverse: 5′-CTT TCC CTC CGC ATT GAC AC-3′; IFN-γ Forward: 5′-ATG AAC GCT ACA CAC TGC ATC-3′, Reverse: 5′-CCA TCC TTT TGC CAG TTC CTC-3′; Foxp3 Forward: 5′-CAC AAT ATG CGA CCC CCT TTC-3′, Reverse: 5′-AAC ATG CGA GTA AAC CAA TGG TA-3′.

### In-vitro Th2 differentiation assays

Naïve CD4^+^ T cells were isolated from 3 DO11.10 mice, and a single-cell suspension was prepared by grinding spleen against a 70-μm nylon cell strainer. After lysis of red cells by red-blood-cell lysis buffer, naïve CD4^+^ T cells (CD4^+^ CD62L^high^ CD25^-^) were first purified by a Mouse CD4^+^ T Cell Isolation Kit (Miltenyi Biotech) and followed by FACS-sorting. Cells were seeded in 48-well plates for further experiments. The seeding density was 2 × 10^5^ naïve T cells/well.

Naïve T cells were maintained in RPMI 1640 (Invitrogen Life Technologies, Carlsbad, CA) supplemented with 2 mmol/L L-glutamine, 10% fetal-calf serum (FCS), 100 mg/ml streptomycin, 100 IU/ml penicillin, 10 mm HEPES, and 20 mm sodium hydrogen carbonate (Invitrogen Life Technologies, Carlsbad, California, USA). The cells were activated with plate-bound anti-CD3 (2 μg/ml), and soluble anti-CD28 (2 μg/ml) antibodies (BD PharMingen). Th-neutral conditions (Th0) contained no exogenous cytokines or anti-cytokines. Th2 conditions contained 10 ng/ml IL-4 (R&D Systems) with 10 μg/ml anti-IFN-γ (BD PharMingen). Where indicated, IL-2 was added at 100 IU/ml. All ATRA (Sigma, St Louis, MO) were dissolved in dimethyl sulfoxide (DMSO) at stock concentrations of 0.01 M and stored at −80°C in light-proof containers. Stocks were thrown away after 4 freeze-thaw cycles. Cultures containing ATRA were protected from light throughout the time of culture; unless stated otherwise, ATRA was used at different concentration from 0.01, 0.05, 0.1, 0.5, 1, 2.5, 5 to 10 μmol/L.

### Statistics

Data are presented as mean ± SEM. The differences between mean values were calculated using student’s *t* test, and a p value of less than 0.05 was considered significant. All experiments were repeated at least 3 times, and n = 10 in each experimental group.

## Abbreviations

ATRA: All-trans retinoic acid; BALF: Bronchoalveolar lavage fluid; AHR: Airway hyperresponsiveness.

## Competing interests

The authors declare that they have no competing interests.

## Authors’ contributions

JW performed experimental studies, carried out the immunoassays and drafted the manuscript. YZ participated experimental studies and analyzed data. QL and WZ performed the statistical analysis and helped to draft the manuscript. ZX conceived of the study, designed experiments, analyzed data, and helped to draft the manuscript. All authors have read and approved the final manuscript.
